# Molecular identification of antagonistic bacteria from Tehran soils and evaluation of their inhibitory activities toward pathogenic fungi

**Published:** 2011-09

**Authors:** AR Ranjbariyan, M Shams-Ghahfarokhi, S Kalantari, M Razzaghi-Abyaneh

**Affiliations:** 1 Department of Mycology, Faculty of Medical Sciences, Tarbiat Modares University, Tehran 14115-331, Iran; 2 Department of Mycology, Pasteur Institute of Iran, Tehran 13164, Iran

**Keywords:** Antifungal activity, Pathogenic fungi, *Bacillus*, 16S rRNA, *Pseudomonas*

## Abstract

**Background and Objectives:**

To find antagonistic bacteria with potential antifungal activity against some pathogenic fungi, including *Aspergillus niger*, *A. flavus, Fusarium moniliforme* and *Penicillium marneffei*, a total of 148 agricultural soil samples from different sites of Tehran were examined.

**Materials and Methods:**

Antagonistic soils were selected by screening against *A. niger* on glucose-yeast extract (GY) agar using a visual agar plate assay method. All growing bacteria were examined for antifungal activity, and antagonistic bacteria identified based on 16S rRNA sequence analysis. Among a total number of 97 bacteria isolated form inhibitory soils (36 samples), 16 bacteria were reported as strong growth inhibitors in co-cultures on GY agar with all tested fungi at variable degrees. Fungal growth inhibitory bacteria were cultured against all fungi and growth inhibition was measured and analyzed between test and control groups by statistical analysis (ANOVA).

**Results:**

Molecular identification of antagonistic bacteria indicated that most bacterial isolates belonged to the genus *Bacillus* (81.25%), including *B. subtilis* (5 isolates), *B. amyloliquefaciens* (6 isolates) and *B. valismortis* (2 isolates)**, followed by one isolate (6.25%) from each *Streptomyces* sp., *Pseudomonas chlororaphis* and *Acinetobacter baumannii*. Based on the visual plate assay results, total fungal growth inhibition of all bacteria was reported in the range of 13.2 to 68.3%. *P. chlororaphis* S105 was reported as the most potent antagonistic bacterium which inhibited the growth of *A. niger* by 68.3%, followed by *F. moniliforme* (66.4%), *A. flavus* (64.7%) and *P. marneffei* (57.1%).

**Conclusion:**

*P. chlororaphis* and some other inhibitory bacteria reported in the present study, they may be considered not only as a rich source of useful metabolites with potential application in antifungal drug discovery, but also as potential candidates for biological control programs.

## INTRODUCTION

The fungal kingdom comprises an estimated 1.5 million species on our planet. Among nearly 100,000 described species of fungi, approximately 400 species is now recognized as pathogens to humans, animals and plants. They include both molds and yeasts from different genera and species with the majority belonging to the genera *Aspergillus*, *Penicillium* and *Fusarium* (1, 2).

The fungi belonging to the genera *Aspergillus, Penicillium* and *Fusarium* are important from the point of not only causing life-threatening infections in humans and animals, but also producing toxic metabolites named “mycotoxins” (3). Among *Aspergillus* species, *A. flavus* is a human pathogen, allergen and mycotoxin producer, while *A. niger* is generally involved in the etiology of otomycosis aside its major role as a plant pathogenic fungus (3). *Fusarium moniliforme* is an important plant pathogenic fungus capable of producing different mycotoxins in food and agricultural commodities (1–3). *Penicillium marneffei* is a saprophytic fungus responsible for opportunistic invasive infections in immunocompromised patients (2).

Synthetic chemicals including antifungal drugs and fungicides are widely used to control detrimental effects of fungi on human health and agriculture. Although fungicides are a key component of disease management programs, they suffer from large limitations including adverse reactions on biological systems, development of resistance by fungal pathogens and undesirable effects on non-target beneficial microorganisms sharing the ecosystem. Thus, there is a clear tendency towards optimization of environmentally-friendly fungicides that produce minimal damage to human health and surrounding ecosystem (4, 5).

The primary current means for the identification of new antifungal agents are represented by screening of the vast biodiversity prevalent in natural resources such as soil samples, marine waters, insects, and tropical plants (6, 7). The need for safe and effective antifungal agents has triggered considerable interest in the isolation of new compounds from biological resources. Likewise, the rapid emergence of fungal pathogens resistant to currently available antibiotics has further compounded the dearth of novel antifungal agents.

Among the existing biodiversity, bacteria have received major consideration not only for their extremely wide distribution and population diversity, but also for their capability to produce a wide array of bioactive metabolites with antimicrobial properties. Nowadays, hundreds of chemically diverse antifungal compounds have been isolated from a vast array of bacteria and there are more compounds waiting to be discovered by researchers yet (8, 9). Since the production of antifungal metabolites in bacteria is quite dependent on the strain and species, ongoing search for finding new bacterial populations to increase the chance of discovering novel antifungals is currently done all over the world.

In the present study, antifungal activities of the soil bacteria identified by the molecular methods were evaluated against some important pathogenic fungi in order to be considered not only as a potential candidate for biological control programs, but also for finding rich sources of useful metabolites with potential application in antifungal drug discovery.

## MATERIALS AND METHODS

**Fungal strains and preparation of inoculums**. *A. niger*, *A. flavus, Fusarium moniliforme* and *Penicillium marneffei* were studied. All cultures were obtained from the Department of Mycology, Pasteur Institute of Iran. For preparing fungal spores, fungal species were cultured on Sabouraud Dextrose Agar (Peptone 1.0%, Glucose 2.0%, Agar-agar 1.5%; E. Merck, Germany) at 28°C for 10 days. Spore suspensions were prepared by gently rubbing the culture surface by glass rod after adding adequate amount of sterile distilled water contained 0.1% Tween 80. The number of fungal spores was determined microscopically with a hemacytometer (10).

**Soil samples and initial screening for antagonistic bacteria**. A total number of 148 soil samples were obtained from different parts of the province of Tehran, Iran. All the samples were transferred to the laboratory in sterile plastic containers. For initial screening of inhibitory soils, 0.1 g of each soil sample was suspended in 0.5 ml 0.9% (w/v) NaCl, mixed by vortex for 30 s, and centrifuged at 2500 rpm for 30 s. Two µl of *A. niger* conidial suspension (2×105 conidia/ml) was spotted onto the center of a GY (Glucose 2.0%, Yeast extract 0.5%, Agar 1.5%) plate, and 10 µl supernatant of each soil sample was placed around the periphery of the plate, 3 cm distance from the *A. niger* spot. Screen plates were incubated at 28°C for 5 days and assessed visually for antifungal phenotypes (10).

**Isolation and screening the bacteria for antifungal activity**. Soil samples with inhibitory activity toward the growth of *A. niger* in initial screening were streaked onto GY agar and incubated at 28°C until bacterial colonies developed. Individual colonies were inoculated onto GY agar and they were screened for antifungal activity as described above. Pure cultures of bacterial isolates that showed antagonistic effects on *A. niger* were further tested for antifungal activity against selected fungi by visual agar plate assay according to Hua *et al*. with some modifications (10). An amount of 10 µl of fungal spore suspension (200 cells/µl) from each fungus was inoculated on GY agar plates in a single streak down the middle of plate. A single streak of each selected bacterium with antifungal activity grown overnight in Tryptic Soy Agar (TSA; Difco, Becton Dickinson, Franklin Lakes, NJ) at 28°C was suspended in 2.0 ml sterile distilled water to obtain a density equal to 0.5× McFarland. Ten µl of each bacterial suspension was inoculated in peripheral lines in distance of 1.5 cm from central line by tooth pick. Triplicate plates were incubated at 28°C for 7 days in static condition. Antifungal activity was assessed by comparing the zone of fungal growth inhibition in fungi co-cultured with bacteria as tests, in comparison with control plates which were inoculated only with corresponding fungi.

**Identification of bacterial isolates**. Antagonistic bacteria were identified by using 16S ribosomal RNA (rRNA) gene sequence analysis. Overnight cultures on LB medium at 30°C were streaked on TSA plates. Single colonies from cultures grown on TSA at 28°C were suspended in 2.0 ml sterile distilled water to obtain a density equal to 0.5× McFarland. Bacterial cells were pelleted by centrifugation at 12,000×g for 10 min. and resuspended in 0.1 ml sterile distilled water. Total DNA was obtained using a DNeasy Plant Mini kit (Qiagen) by following the supplier's instructions. 16S rRNA gene fragments were amplified by polymerase chain reaction (PCR) using 1 µl of each cell suspension as template and universal primers *27F* (5′-AGAGTTTGATCMTGGCTCAG-3′) and *1525R* (5′AAGGAGGTGWTCCARCC-3′) (11). The PCRs were carried out using approximately 500 ng of total bacterial DNA, 10 µl of 10x PCR buffer, 8 µl of MgCl_2_ (25 mM), 10 µl of deoxynucleoside triphosphates (dNTPs) (2 mM each), 3.3 µl of each primer (20 µM), 0.5 µl of *Taq* polymerase (5 U/µl), and enough Milli Q water so that the final volume of the mixture was 100 µl. The PCR mixtures were denatured at 95°C for 5 min, which was followed by 35 cycles of 94°C for 30 s, 58°C for 30 s, and 72°C for 70 s and then a final extension at 72°C for 5 min. Amplification was checked for purity by electrophoresis on a 1.0% agarose gel. The bands of interest were excised from the gel, and the DNA was purified using QIAquick PCR purification columns (Qiagen, Inc., Valencia, CA). Purified DNA fragments were sequenced using the same sets of primers that were used for amplification by an ABI Prism BigDye® Terminator v3.1 Cycle Sequencing Kit (Applied Biosystems). Bacteria were identified based on sequence similarities to homologous 16S rRNA gene fragments in the Ribosomal Database Project database (12) (accessed at http://rdp.cme.msu.edu/ index.jsp). For biochemical identification, bacteria were first determined to be either Gram-positive or Gram-negative using potassium hydroxide (13). Gram-positive isolates were identified using GP2 MicroPlates (Biolog), whereas Gram-negative isolates were identified using GN2 MicroPlates (Biolog), according to the instructions of the manufacturer. Identification was based on the similarity index of carbon source utilization by each isolate relative to that of identified reference strains in the Biolog GP and GN databases.

**Statistical Analysis**. Quantitative data of fungal growth were analyzed by under windows SPSS version 16 program. The *P<*0.05 was considered significant.

## RESULTS

**Fig. 1:**
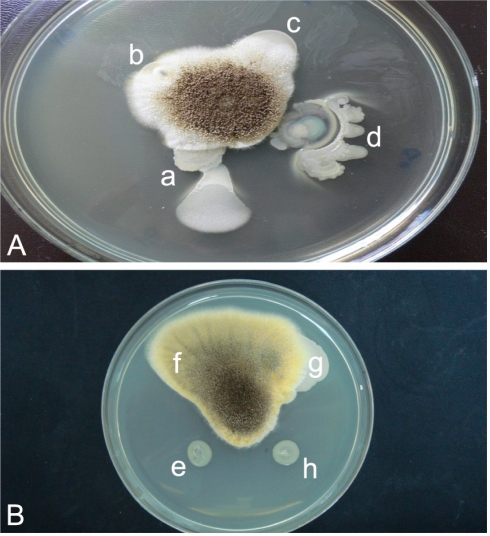
Screening of soil samples and isolated bacteria for antagonistic activity against *A. niger* on GY agar. Fig. 1A shows inhibitory (a & d) and non-inhibitory (b & c) soils, while Fig. 1B shows bacterial isolates inhibitory (e & h) and non-inhibitory (f & g) for fungal growth.

**Initial screening of inhibitory soils and bacteria**. Among 148 soil samples screened for antifungal activity against *A. niger*, 36 soils were found to be inhibitory for the fungus in GY plates. Fig. 1A shows inhibitory (a and d) and non-inhibitory (b and c) soils in initial screening against *A. niger*. Bacterial antagonism is visible as a clear zone of fungal growth inhibition around the isolates “a” and “d” from soil no. 75. A total number of 97 bacteria were isolated from inhibitory soils were classified according their degree of antagonism into three groups including i) strong (16 isolates), ii) moderate (32 isolates) and iii) no effect (49 isolates) based on visual agar plate assay on GY agar. Fungal growth inhibition by individual pure bacteria is shown in Fig. 1B as a clear zone of fungal growth retardation surrounding the bacterial colonies e and h in comparison with non-inhibitory bacteria i.e. isolates f and g.

**Identification of antagonistic bacteria**. Identification of antagonistic bacteria (group i) selected by visual agar plate assay on GY agar was carried out by 16S ribosomal RNA (rRNA ∼1 kb) sequencing using ABI prism big Dye terminator cycle sequencing apparatus. PCR was carried out by using primers *27F* and *1525R* and FASTA search of the GenBank database was used for identification of the isolates at species level. General characteristics of the identified bacteria are summarized in Table 1. They included *Bacillus amyloliquefaciens* (6 isolates), *B. subtilis* (5 isolates), *B. valismortis* (2 isolates) and one isolate of each *Acinetobacter baumannii*, *Streptomyces* sp. and *Pseudomonas chlororaphis*. Regard to the antagonistic phenotypes of isolated bacteria, it was shown that all the bacteria had both zone of growth inhibition (zi) and contact growth inhibition (ci) for fungi tested except *A. baumannii* S1, *B. subtilis* S105 and *P. chlororaphis* S105 which showed only zi for fungal growth.

**Table 1:** General features of soil bacteria with strong inhibitory activity toward the growth of tested fungi.

Genus	Species	Isolate	Gram staining	Antagonistic phenotype^a^
*Acinetobacter*	*A. baumannii*	S1	-	zi
	*B. subtilis*	S2	+	zi/ci
	*B. subtilis*	S23	+	zi/ci
	*B. subtilis*	S105	+	zi
	*B. subtilis*	S115	+	zi/ci
	*B. subtilis*	S140	+	zi/ci
	*B. amyloliquefaciens*	S23	+	zi/ci
*Bacillus*	*B. amyloliquefaciens*	S85	+	zi/ci
	*B. amyloliquefaciens*	S102	+	zi/ci
	*B. amyloliquefaciens*	S123	+	zi/ci
	*B. amyloliquefaciens*	S124	+	zi/ci
	*B. amyloliquefaciens*	S143	+	zi/ci
	*B. valismortis*	S24	+	zi/ci
	*B. valismortis*	S121	+	zi/ci
*Pseudomonas*	*P. chlororaphis*	S105	–	zi
*Streptomyces*	*Unknown*	S40	+	zi/ci

a Phenotypes of co-cultures on GY agar. zi: zone of growth inhibition; ci: contact growth inhibition.

**Fig. 2:**
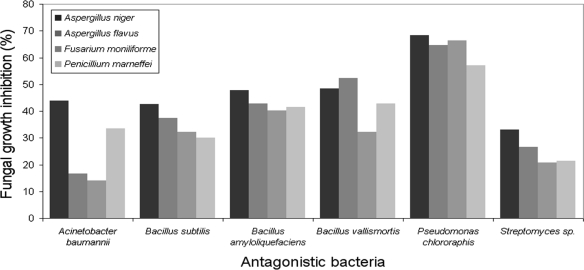
Comparative results of the quantitative assay of the antifungal activity of identified soil bacteria in agar plate assay on GY agar. Results are from the means±SEM of two separate experiments in triplicate sets for total bacteria in each species.

**Antifungal activity against tested fungi**. All 16 identified bacteria were tested for potential antifungal activities against some selected fungi visually by comparing fungal growth in fungi co-cultured with bacteria relative to controls inoculated only with fungi. Antagonistic activity of selected bacteria against all tested fungi which was presented as mean zone of fungal growth inhibition (Table 2). The growth of all tested fungi was inhibited by all selected bacteria by different extents and inhibition was significant for the majority of fungi in comparison with relative controls (ANOVA, *P<*0.05). Comparative results of the quantitative assay of the inhibitory activity of identified soil bacteria against selected fungi are shown as mean of growth inhibition for each bacterial species in Fig. 2. Antifungal activities of *A. baumannii*, *B. subtilis*, *B. amyloliquefaciens*, *B. valismortis*, *P. chlororaphis*, and *Streptomyces* sp. isolates against tested fungi were reported in the range of 14.1-43.9%, 13.2-62.7%, 24.6-66.0%, 25.7-60.0%, 57.1-68.3% and 20.7-33.0%, respectively. *P. chlororaphis* S105 was reported as the most potent antagonistic bacterium which inhibited the growth of *A. niger* by 68.3%, followed by *F. moniliforme* (66.4%), *A. flavus* (64.7%) and *P. marneffei* (57.1%).

**Table 2:** Inhibitory effects of antagonistic bacteria against some opportunistic fungi on GY agar.

Genera	species	% of fungal growth inhibition for:			
		
		*A. niger*	*A. flavus*	*F. moniliforme*	*P. marneffei*
*Acinetobacter*	*A. baumannii* S1	43.9*	16.8	14.1	33.6*
*Bacillus*	*B. subtilis* S2	52.8*	31.3*	44.5*	35.7*
	*B. subtilis* S23	35.3*	49.0*	25.8*	26.4*
	*B. subtilis* S105	62.7*	39.0*	49.2*	26.3*
	*B. subtilis* S115	13.2	35.7*	18.0	28.5*
	*B. subtilis* S140	49.5*	32.5*	24.6*	33.6*
	*B. amyloliquefaciens* S23	33.0*	35.9*	24.6	28.6*
	*B. amyloliquefaciens* S85	42.9*	34.8*	40.4*	40.7*
	*B. amyloliquefaciens* S102	48.5*	32.3*	44.1*	45.7*
	*B. amyloliquefaciens* S123	66.0*	60.1*	55.8*	45.5*
	*B. amyloliquefaciens* S124	65.0*	62.3*	49.2*	42.8*
	*B. amyloliquefaciens* S143	30.7*	32.4*	28.5*	45.6*
	*B. vallismortis* S24	51.8*	44.7*	39.1*	40.7*
	*B. vallismortis* S121	45.2*	60.0*	25.8	42.9*
*Pseudomonas*	*P. chlororaphis* S105	68.3*	64.7*	66.4*	57.1*
*Streptomyces*	*Streptomyces sp* S40	33.0*	26.7*	20.7*	21.4*

^a^The data are the mean±SEM of two separate experiments in triplicate sets.

^b^Asterisks show statistically significant difference with a control (*P<*0.05).

^c^Control cultures (non-exposed to the bacteria) for *A. niger, A. flavus, F. moniliforme* and *P. marneffei* had mean growth rates of 30.3, 30.0, 25.6 and 14.0 mm, respectively.

## DISCUSSION

It has been reported that, on average, two or three antibiotics derived from bacteria enter the market each year (9). Likewise, bacterial-based bio-control products have now been commercially developed for control of fungal spoilage of food products and agricultural commodities (14). It has been shown that production of an extremely wide array of bioactive compounds by bacteria and their potential for use in biocontrol programs is completely dependent on parameters such as taxonomical position and physiological characters (i.e. species, varieties and growth cycle), geographic condition, soil composition, etc. So, screening of a large number of bacteria from different geographic locations may increase the chance of finding novel bioactive compounds with broad spectrum antifungal activity.

In the present study, to maximize the chance for isolating bacteria suitable as rich sources of antifungal bioactive metabolites as well as for potential development of biological control agents for use against fungi, we isolated and screened bacterial populations from Tehran soil samples. A number of 16 antagonistic bacteria with strong inhibitory activity against selected fungi were isolated from 148 soil samples and identified in the genera *Bacillus*, *Pseudomonas*, *Acinetobacter* and *Streptomyces* using 16S rRNA sequence analysis. Inhibitory bacteria with antifungal activity against some selected fungi were isolated from approximately 25.0% of soil samples of which about 16.5% were reported as strong inhibitors of fungal growth in bioassays. They were identified in the genera *Bacillus* (*B. subtilis*, *B. amyloliquefaciens* and *B. valismortis*), *Acinetobacter* (*A. baumannii*), *Pseudomonas* (*P. chlororaphis*) and *Streptomyces* (an unidentified species). All these species exhibited considerable inhibitory activity toward the growth of all tested fungi in variable degrees which indicate that population diversity is an extremely important factor determining the potential for antagonistic activity of bacteria toward various microorganisms. Our data indicated reliability of GY agar as a suitable low cost and easy screening test for evaluating antagonistic activities of a large number of bacteria against fungi from different genera.

As reported by other researchers, the genera *Bacillus*, *Pseudomonas*, *Agrobacterium* and *Streptomyces* have been considered as main bacterial genera capable of producing antifungal bioactive metabolites (15, 16). In the present study, the genus *Bacillus* was the most important antagonistic bacterial group which comprised around 80.0% of the inhibitory isolates. Within *Bacillus* species, *B. subtilis* as the most important species and in some extents other species such as *B. amyloliquefaciens* and *B. valismortis* are reported to produce a wide range of structurally related antimicrobial compounds and they are usually isolated from the soil as the main natural habitat (17–20). Among active compounds, with amazing variety of structures produced by *B. subtilis*, peptide antibiotics represent the predominant class. The iturin family contains the closely related cyclic lipopeptides the iturins, mycosubtilins and bacillomycins with anti-fungal activities (15, 17). Members of the genus *Pseudomonas* also comprise a large group of the active biocontrol strains as a result of their general ability to produce a diverse array of potent antifungal metabolites. Antibiotic production by fluorescent pseudomonads was recognized as an important feature in plant disease suppression by some strains. These include simple metabolites such as 2,4-diacetylphloroglucinol, phenazine-1-carboxylic acid and pyrrolnitrin [3-chloro-4-(2’-nitro-3’-chlorophenyl)-pyrrole], as well as the complex macrocyclic lactone, 2,3-de-epoxy-2,3-didehydrarhizoxin. Pyrrolnitrin is active against *Rhizoctonia* spp, *Fusarium* spp, and other phytopathogenic fungi, and it has been used as a model in the development of a new phenylpyrrole agricultural fungicide (21–23). The strong inhibitory activity of *Bacillus* species as well as other bacteria, specially *A. baumannii* and *P. chlororaphis* isolated in the present study, may be attributed to production of antifungal peptides by *Bacillus* strains (15–20) and production of aerugine, phenazine, and phloroglucinol antibiotics by *Pseudomonas* species (21–23).

With respect to the considerable tolerance of *B. subtilis* and *P. chlororaphis* to environmental stresses and their facile production by current fermentation technology, bacterial isolates identified in this study with a diverse range of antifungal activities may be considered as potential sources of novel antifungal bioactive metabolites as well as promising candidates to develop new biocontrol agents for controlling pathogenic fungi in medicine and agriculture. We have demonstrated that *P. chlororaphis* S105 may produce antifungal compounds suitable to control the fungal diseases as a promising eco-friendly tool. Future studies to identify antifungal metabolites of antagonistic bacteria isolated here, to determine their mechanisms of action on fungal cells and their evaluation as effective fungal biocontrol agents in the field are recommended.
